# Postshift Hand Fatigue in Emergency Service Nurses: Relationships Between Different Functional Tests and Perceived Effort Scores

**DOI:** 10.1155/2024/8835383

**Published:** 2024-11-04

**Authors:** Fadime Ulupınar, Sibel Meler, Şeyda Karasu, Süleyman Ulupınar

**Affiliations:** ^1^Erzurum Technical University, Faculty of Health Sciences, Department of Nursing, Erzurum, Turkey; ^2^Faculty of Medicine Hospital, Selçuk University, Konya, Turkey; ^3^Department of Coaching Education, Faculty of Sport Sciences, Erzurum Technical University, Erzurum, Turkey

## Abstract

**Objective:** Hand functionality, defined as the ability to perform tasks requiring fine motor skills, is crucial for emergency service nurses as it directly affects their ability to perform tasks requiring fine motor skills, such as administering medication, operating equipment, and providing patient care. This study, therefore, aimed to investigate the effects of an 8-h work shift on hand functionality and perceived exertion among emergency service nurses.

**Method:** Employing a cross-sectional design, the study measured manual dexterity, handgrip, and pinch strength, and perceived exertion using the Minnesota Manual Dexterity Test (MMDT), Nine-Hole Peg Test (9-HPT), Handgrip Strength Test, Pinch Strength Test, and the Borg Rating of Perceived Exertion (RPE) Scale. Data were collected from 34 emergency service nurses both before and after their shifts.

**Results:** The present findings indicated significant postshift declines in manual dexterity as evidenced by the MMDT and 9-HPT, with the former demonstrating a large effect size and the latter a small effect size. No significant changes were observed in handgrip and pinch strength. Notably, Borg RPE scores increased significantly postshift, indicating substantial perceived fatigue with a nearly perfect effect size. Correlation analyses revealed significant relationships between increased physical exertion and changes in hand dexterity and strength, underscoring the physical demands placed on nurses during typical work shifts.

**Conclusions:** These findings highlight the need for healthcare institutions to reassess work schedules and ergonomic practices to mitigate fatigue and preserve nurses' hand functionality, thereby enhancing patient care and nurse well-being. The study calls for further research to explore more comprehensive strategies aimed at reducing the occupational strain on emergency service nurses.

## 1. Introduction

Emergency departments (EDs) are among the most critical and demanding environments in healthcare services, characterized by the need for swift and effective responses to constantly changing patient needs [1, 2]. Nurses operating in these intense settings are subjected to long working hours, physically and mentally challenging tasks, emergencies, and unforeseen events, contributing to their daily workload [3]. Such conditions not only elevate stress levels but also lead to significant physical fatigue, particularly affecting hand and upper extremity function [4, 5]. Over time, the relentless pace and demands of this work can cause serious fatigue and functional impairments, endangering both the health of the nurses and the safety of their patients [6, 7].

Fatigue has been shown to significantly alter the coordination of muscle activity, leading to functional imbalances that can increase the risk of operational errors in tasks requiring fine motor skills [8]. This is particularly concerning in high-stress environments like EDs, where nurses rely heavily on precise hand movements for tasks such as administering medication, operating medical devices, and providing patient care. Additionally, research has demonstrated that muscle fatigue impairs coordination and increases variability in muscle activation patterns, which may further exacerbate the risk of errors during prolonged shifts [9, 10]. Therefore, a thorough examination of the workload and fatigue experienced by emergency service nurses is crucial for addressing these issues and enhancing working conditions.

In nursing practice, particularly within the high-intensity environment of EDs, the role of hands is fundamental [11]. Nurses rely on their hands for a wide range of tasks, including operating medical devices, preparing medications, administering injections, and assisting patients physically [11, 12]. The relentless use of hands, exacerbated by lengthy shifts, can precipitate hand fatigue and lead to musculoskeletal disorders over time [13, 14]. Mechanisms proposed in the literature suggest that muscle fatigue results from a reduction in force production capability, which impairs motor performance and increases the risk of repetitive strain injuries [10]. Additionally, the extensive use of handheld devices has been associated with musculoskeletal disorders due to the repetitive use of specific hand muscles, leading to conditions such as tendinitis and myofascial pain syndrome [15]. Moreover, work-related musculoskeletal disorders of the hand and wrist are exacerbated by repetitive, hand-intensive movements, which can cause tissue inflammation, nerve injury, and sensorimotor changes that further impair hand function [16]. These mechanisms collectively underscore the importance of addressing fatigue-related risks and ergonomic challenges in high-stress work environments.

The objective assessment of hand fatigue is paramount for maintaining nurses' health and sustaining their job performance [13, 17]. To this end, a variety of tests are employed to evaluate hand fatigue and functional capacity [17, 18]. However, it is crucial that these tests are both valid and reliable to ensure they accurately measure the intended attributes and yield consistent outcomes [19–21]. Therefore, these assessments are designed to provide a thorough evaluation of the hand functions that are essential for nurses to perform their duties effectively. In this study, four distinct functional tests were meticulously selected to evaluate the hand fatigue and hand functions of nurses from various perspectives. The Minnesota Manual Dexterity Test (MMDT) assesses participants' hand–eye coordination and fine motor skills, mirroring nurses' abilities to manipulate medical devices and execute precise operations [22]. More specifically, the Nine-Hole Peg Test (9-HPT) measures the efficiency with which nurses can use their hands and fingers, a crucial skill for performing daily nursing tasks [23]. The Handgrip Strength Test gauges the strength of handgrip, reflecting nurses' ability to support patients or transport heavy medical equipment [24]. Lastly, the Pinch Strength Test evaluates the force of the fingertips, essential for activities that demand fine motor skills, such as administering injections or managing small items [20]. Each test targets distinct elements of hand fatigue, and collectively they offer a thorough assessment of overall hand function. This diversity underscores the complex nature of hand fatigue and the range of hand use within nursing practice.

In addition to functional tests, the Borg Rating of Perceived Exertion (RPE) Scale offers nurses a means to subjectively assess their levels of physical effort and fatigue, thus facilitating an exploration of the relationship between objective measures of hand fatigue and individual perceptions [25, 26]. The Borg RPE Scale allows nurses to self-report their overall fatigue level at the end of their shifts on a scale ranging from 6 to 20, enabling a clearer identification of how physical fatigue levels affect functional hand skills. Understanding the correlation between data from the MMDT, 9-HPT, Handgrip Strength Test, and Pinch Strength Test and nurses' perceived levels of fatigue can uncover the immediate effects of hand fatigue on nursing performance. Consequently, this study was designed to comprehensively assess the impact of an 8-h work shift on hand fatigue and perceived exertion among emergency room nurses, while also determining which specific hand functions are most significantly impacted by such shifts and examining the interrelationships among these functions. Therefore, objective data from the MMDT, 9-HPT, Handgrip Strength Test, and Pinch Strength Test were integrated with subjective fatigue evaluations using the Borg RPE to illustrate the complex nature of hand fatigue.

## 2. Methods

### 2.1. Study Design

**Figure 1:**
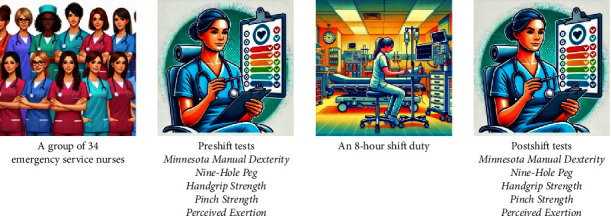
Research design.

This study employed a repeated measures design to evaluate the hand fatigue and perceived exertion levels of emergency service nurses before and after their shifts (Figure 1). This design was chosen because it allows for the direct comparison of each nurse's performance at two different time points, thus controlling for individual differences that might affect the results. By measuring the same participants both preshift and postshift, we could more accurately assess the impact of an 8-h shift on hand functionality and exertion levels. This approach enhances the internal validity of the results by reducing the variability that could arise from between-subject differences.

Hand fatigue was quantified using four distinct tests: the MMDT, 9-HPT, Handgrip Strength Test, and Pinch Strength Test. In addition, nurses self-assessed their levels of physical fatigue at the beginning and end of their shifts using the Borg RPE Scale. The selected assessments, comprising the MMDT (approximately 5 min), 9-HPT (approximately 3 min), Handgrip Strength Test (approximately 2 min), Pinch Strength Test (approximately 3 min), and Borg RPE Scale (approximately 1 min), required about 15 min per participant for each measurement point. Consequently, conducting these tests both preshift and postshift resulted in a total estimated duration of approximately 30 min per participant.

### 2.2. Participants

Utilizing G ∗ Power statistical software and focusing on the difference between two paired means (pretest–posttest for this study), a minimum sample size of 34 individuals was determined. This calculation assumed a medium effect size (*d* = 0.50), 80% power level, and a 5% significance level (two-tailed) [27]. The participants were registered nurses aged between 20 and 40 years, all of whom provided direct patient care in EDs. These limits were selected to focus on nurses who are actively engaged in the demanding tasks of emergency nursing while minimizing the potential confounding effects of age-related declines in fatigue resistance and hand–eye coordination that may occur in those over 40. This choice allows us to control for age-related variables that might influence the study's outcomes. Although this age range limits the generalizability of the results to nurses older than 40 years, the findings suggest that if risks are present in the 20–40 age group, these risks may be even more pronounced in older nurses [28, 29]. Consequently, the results of this study could be considered conservative estimates of the potential risks faced by nurses over 40 years of age. Eligibility for the study required at least 1 year of job experience and good physical health. Excluded from the study were non-nursing health professionals, personnel not directly involved in patient care, individuals with upper extremity health problems, those with a history of psychiatric or cardiovascular conditions, and anyone taking specific medications such as antidepressants, tranquilizers, hypnotics, anti-Parkinson drugs, or glucose-lowering drugs. Participants were instructed to maintain their usual dietary habits to minimize any dietary influences on the study results. Entry into the study was contingent upon voluntary participation and the signing of an informed consent form, ensuring adherence to ethical standards.

### 2.3. MMDT

The MMDT MMDT was employed to evaluate the participants' hand skills and speed, serving as a comprehensive measure of their hand–eye coordination and fine motor abilities [22]. The MMDT required participants to rapidly place colored disks into corresponding slots on a standardized pegboard, a task designed to assess manual dexterity. Participants were seated at a table, with adjustments made to ensure the pegboard was positioned at a comfortable height for optimal performance. The test involved a pegboard furnished with 60 round holes, organized into four horizontal rows, which accommodated the placement and manipulation of the disks. The disks used for the test were specifically designed for this purpose, each measuring 3.7 cm in diameter, 1.8 cm in height, and weighing 15 g. During the evaluation, participants were instructed to use their dominant hand to pick up each disk, turn it over, and then accurately place it back into its respective hole. Each participant's time to complete the task was meticulously recorded in seconds. The faster completion time indicates better manual dexterity and hand–eye coordination.

### 2.4. 9-HPT

The 9-HPT, employing a commercial plastic setup, assessed the hand functions of the participants. This task involves rapidly inserting and removing nine pegs into matching holes [23]. The 9-HPT kit features a board of dimensions 31 × 26 × 4 cm, equipped with nine pegs, each 0.6 cm in diameter. For the test, the pegboard was aligned with each participant's midline, with participants seated to ensure the board's surface was level with their chest. Participants were instructed to sequentially insert and then extract the pegs as swiftly as possible using their dominant hand, without a predefined order. Scoring relied on the aggregate time to accomplish the task, recorded in seconds, starting when the first peg was picked up and ending as the final peg was placed.

### 2.5. Handgrip Strength Test

In this study, participants' grip strength was assessed using the Handgrip Strength Test, recognized as a reliable measure of overall hand strength and an indicator of [24]. This evaluation was conducted with a commercial hand dynamometer (Takei A5001 Hand Grip Dynamometer). Participants used their dominant hand to grasp and exert maximum effort on the device. They performed this action twice, allowing for a rest period of 2–3 min between attempts to prevent muscle fatigue from affecting the results. The highest force exerted in each of the two trials was recorded as the individual's handgrip strength. Proper posture was maintained throughout the test, and participants were instructed to apply force to the dynamometer in a standardized manner to ensure the reliability and comparability of the results.

### 2.6. Pinch Strength Test

The Pinch Strength Test was administered to evaluate participants' pinch force, a practical measure of fine motor skills and gripping capacity [20]. Designed to assess the impact of hand fatigue on these capabilities, the test encompassed the tip-to-tip grip position. Using a commercial force gauge (Jamar Hydraulic Pinch Gauge), participants applied maximum force in this grip style. They performed two attempts, with adequate rest between sessions, and the higher measurement was recorded as the participant's valid pinch force.

### 2.7. Borg RPE Scale

The Borg RPE Scale was utilized to assess the perceived effort and fatigue levels among emergency service nurses. This scale operates on the principle of self-assessment, where individuals rate their fatigue and strain levels during physical exertion on a scale from 6 to 20 [25, 26]. It provides a means for participants to subjectively report their physical condition, albeit within a standardized context. In this study, nurses were requested to indicate their current levels of fatigue using the Borg RPE at two distinct times: before starting their shift and after completing it. Employing this scale is vital for evaluating the effects of physical and mental fatigue on nursing performance, offering key insights into how work-related exertion impacts overall well-being and job efficiency.

### 2.8. Statistical Analysis

Statistical analyses were conducted using IBM SPSS for Windows, Version 25.0, Armonk, New York, USA. The data were reported as mean ± standard deviation (SD). Differences between pretest and posttest scores (before and after the shift) were evaluated using paired *t*-tests, with a *p* value of less than 0.05 deemed indicative of statistical significance. Furthermore, the effect sizes for changes observed before and after shifts were calculated employing Cohen's d [30] and were categorized based on the Hopkins scale [31, 32]. Additionally, correlation analyses were performed to examine the relationships among the results from the MMDT, 9-HPT, Handgrip Strength Test, Pinch Strength Test, and Borg RPE scores.

## 3. Results

Table 1 assesses the shift impact on manual dexterity and perceived fatigue among emergency service nurses. The MMDT and 9-HPT reveal significant postshift performance declines, highlighting a large and small effect size, respectively. Meanwhile, changes in handgrip and pinch strength were not statistically significant, showing trivial effect sizes. Notably, Borg RPE scores significantly increased postshift, indicating a nearly perfect effect size and underscoring substantial perceived fatigue increases.

Table 2 presents a correlation matrix displaying the relationships between various hand function tests conducted preshift among emergency service nurses. A significant positive correlation was found between the MMDT and the 9-HPT scores, indicating that as one score increases, so does the other. Similarly, a strong correlation was noted between handgrip strength and pinch strength, highlighting a significant relationship in these measures of hand function. On the other hand, both strength scores (handgrip and pinch) showed trivial correlations with both MMDT and 9-HPT scores.

Table 3 illustrates the relationships between percentage changes in hand function tests and perceived exertion among emergency service nurses. Significant correlations were observed between the Borg RPE percentage change and percentage changes in the MMDT, 9-HPT, and handgrip strength, indicating a relationship between increased physical exertion and changes in hand dexterity and strength. The correlation between Borg RPE percentage change and pinch strength percentage change was positive but not significant, suggesting a weaker relationship in this aspect.

## 4. Discussion

This study sought to explore the effects of an 8-h work shift on hand functionality and perceived exertion among emergency service nurses, concentrating on the intense physical demands of emergency care environments. Our findings indicate that a standard work shift can significantly impair hand–eye coordination and manual dexterity, as demonstrated by the MMDT and 9-HPT results. These results are consistent with a growing body of evidence that highlights the negative impact of shift work on cognitive and motor performance among healthcare professionals. For instance, a study reported that shift work was associated with significant declines in cognitive functions such as visual attention, processing speed, and task switching—skills closely related to manual dexterity and hand–eye coordination [33]. Similarly, another research emphasized the widespread impact of fatigue among nurses working extended hours, which often leads to errors in clinical practice and increased risks to patient safety [34]. This aligns with our findings that standard work shifts can substantially impair hand functionality, which is crucial for the precision required in nursing tasks.

Moreover, the challenges faced by aging nurses further underscore the importance of addressing the physical and cognitive demands placed on healthcare workers [35]. As nurses age, their ability to maintain manual dexterity and cognitive performance may decline, exacerbating the risks associated with shift work. These studies collectively reinforce the urgent need for healthcare institutions to reassess work schedules and implement supportive measures, such as ergonomic improvements and fatigue management programs, to alleviate the burden on healthcare professionals and ensure high standards of patient care. Building on these insights, research by Merchaoui et al. [17] has documented the negative effects of prolonged exposure to excessive workloads on healthcare professionals, particularly pointing out a marked decline in cognitive performance among nurses with long career tenures [17]. This cognitive decline is, in part, attributed to fatigue that impairs manual dexterity and essential cognitive functions, which are crucial for maintaining nursing efficiency and safety. Our findings contribute to this broader understanding by highlighting the specific impacts of an 8-h work shift on hand–eye coordination and manual dexterity among emergency service nurses, particularly within the 20–40 age range. This focus underscores the potential severity of these impacts on older nurses, whose cognitive and motor functions may be even more vulnerable to the effects of shift work, thereby suggesting that the risks identified in our study could be more pronounced in a broader, more age-diverse nursing population.

In alignment with these findings, Baker and Roche [36] have highlighted the crucial role of manual dexterity in nursing, stressing its necessity for performing essential tasks such as intravenous drug administration, physical assessments, and catheter insertion. These tasks rely heavily on fine motor skills and coordination, which our study shows to be adversely affected by a typical workday. Baker and Roche suggest methods to enhance manual dexterity among nursing students, such as using all surfaces of the hands and securing objects between fingers, which could also be beneficial for practicing nurses, especially those facing the physical demands of extended work hours. Their recommendation to integrate dexterity training into nursing curricula supports our suggestion that healthcare facilities should conduct regular assessments and implement targeted interventions to counteract the adverse effects of typical work shifts on hand function [36]. Such proactive measures could not only improve the well-being of nursing staff but also enhance the overall quality of patient care in these high-stress environments.

The work environment plays a pivotal role in contributing to nurse fatigue, underscored by a complex array of factors including workload, ergonomics, and psychological stress [34, 37, 38]. Ergonomic interventions, such as adjusting the height of workstations, providing supportive seating, and optimizing the layout of essential tools and equipment, could significantly reduce the physical strain on nurses, thereby mitigating the hand fatigue observed in this study [39, 40]. Additionally, implementing workload management strategies, such as rotating shifts to prevent prolonged repetitive tasks and ensuring adequate staffing levels, may help in distributing physical exertion more evenly among staff, reducing the cumulative stress on hand function [17, 33, 35]. Psychological support programs and stress management training could also alleviate the mental fatigue that contributes to physical exhaustion, further reducing the incidence of hand fatigue [41–43]. The integration of these environmental and organizational changes is crucial for preserving nurses' hand functionality and overall job performance, particularly in high-stress environments like EDs.

Research by Dewanti, Jingga, and Wahyudiono [44] supports our findings, showing the significant impacts of these variables on hand function and perceived exertion among emergency service nurses [44]. These findings advocate for comprehensive interventions that extend beyond scheduling adjustments to include improvements in the physical conditions of the work environment, aiming to reduce fatigue and safeguard the physical and cognitive health of nurses. In conjunction with this, the study by Taylor, Bennett, and Cameron [45] illustrates the evolving dynamics within EDs in Melbourne, Australia, highlighting how external pressures like access blocks and increasing patient numbers have necessitated a shift toward more diversified and expanded care models [45]. This evolution has introduced new services and initiatives designed for more efficient patient management, which may indirectly exacerbate the physical and cognitive demands on emergency service nurses, linking back to the changes we observed in our study. Furthermore, findings by Wingler and Keys [38] delve into how the physical healthcare environment impacts nurse fatigue. They identified 27 design elements that play a critical role in either exacerbating or mitigating fatigue levels. This insight is particularly relevant to our findings, reinforcing the necessity for comprehensive strategies that address not only work schedules but also environmental factors to enhance nurse well-being, patient safety, and care quality—fundamental objectives within nursing management and healthcare administration [38].

An additional layer of complexity is provided by Alshammari et al. [46], whose investigation into fatigue levels among ED nurses in Saudi Arabia highlights the global nature of this issue. Their research shows that work-related fatigue significantly compromises nurses' performance and decision-making capabilities, resonating with the concerns our study raises about the strains of standard work shifts [46]. The identification of demographic predictors of fatigue such as gender, work experience, and the number of dependents, along with psychosocial factors like emotional demand, stress, and burnout, points to the multifaceted nature of nurse fatigue. These findings underline the need for a holistic approach to managing nurse well-being that considers both the physiological impacts of work shifts and the broader psychosocial dimensions of the nursing work environment. This comprehensive perspective is essential for developing effective interventions that improve the overall health and efficiency of nurses in various healthcare settings.

The role of technological advancements and ergonomic design in reducing nurse fatigue has been increasingly recognized, aligning closely with our findings that an 8-h work shift can significantly impair hand–eye coordination and manual dexterity among emergency service nurses. The integration of ergonomic tools and technological aids could significantly alleviate the workload of nurses in these settings and preserve their hand functionality, as our study suggests [47, 48]. Previous studies demonstrate that the adoption of technology not only reduces work-related stress but also enhances job efficiency, which is crucial in maintaining manual dexterity and reducing physical strain—key issues identified in our research [49, 50]. Our results corroborate this need, as we observed a notable increase in the subjective perception of exertion among nurses, indicating that expanding these programs could not only improve mental well-being but also patient care outcomes, reinforcing the interconnectedness of nurse welfare and quality healthcare delivery. Moreover, the importance of education and continuous learning in nursing cannot be overstated [49, 51]. Current investigations show how training modules and simulation-based learning, especially in emergencies, not only enhance nurses' practical skills but also bolster their decision-making abilities under stress [48, 49]. Such educational initiatives are crucial for preventing the decline in manual skills that can occur following intense work shifts, thus supporting nurses in maintaining high standards of patient care even during demanding periods—reflecting similar challenges identified in our study, which highlighted the detrimental effects of standard work shifts on hand functionality and overall nurse performance.

### 4.1. Limitations

This study, while providing valuable insights into the effects of an 8-h work shift on hand functionality and perceived exertion among emergency service nurses, has several limitations that should be considered. First, the sample size and demographic scope of the participants may limit the generalizability of the findings. The participants were predominantly from a single geographical location, which may not accurately reflect the diverse conditions and experiences of nurses globally. Secondly, the study's design was cross-sectional, which restricts the ability to infer causal relationships between work shifts and the observed changes in hand functionality and perceived exertion. While our study provides a snapshot of the effects of an 8-h work shift on these variables, it does not account for how these effects might evolve over time or whether they result directly from the work shifts. A longitudinal design, by contrast, would allow for the observation of changes in hand functionality and perceived exertion over extended periods, potentially providing stronger evidence for causal relationships. By tracking the same participants across multiple time points, a longitudinal study could better isolate the impact of work shifts from other confounding factors and offer more robust insights into the long-term effects of prolonged work schedules on nurses' health and job performance. Additionally, the reliance on self-reported measures for perceived exertion might introduce subjective bias, affecting the accuracy of the data. Although the Borg RPE scale is a validated tool, the subjective nature of self-reporting can vary significantly between individuals. Lastly, environmental factors within the EDs, such as equipment ergonomics and departmental support structures, were not extensively examined but could significantly influence the outcomes. Future research should consider these variables to provide a more comprehensive understanding of the factors contributing to nurse fatigue and manual dexterity impairment.

## 5. Conclusions

This study comprehensively evaluated the impact of an 8-h work shift on manual dexterity and perceived exertion among emergency service nurses. The findings reveal that such shifts significantly impair manual dexterity as measured by the MMDT and the 9-HPT, with the former showing a large effect size and the latter a small effect size. These results suggest that prolonged work can diminish fine motor skills essential for effective nursing. Conversely, changes in handgrip and pinch strength did not show statistical significance, indicating that these aspects of hand function might be less susceptible to the effects of a single work shift. However, a notable increase in Borg RPE scores was observed postshift, highlighting a substantial increase in perceived fatigue, with nearly perfect effect sizes. This suggests that while some aspects of physical strength remain stable over a typical shift, the overall sense of exertion and fatigue increases significantly. Furthermore, the study uncovered significant correlations between manual dexterity tests and perceived exertion, indicating that decreases in dexterity are closely related to increases in fatigue. In conclusion, the study underscores the critical need for healthcare facilities to address the ergonomics of work schedules and to implement targeted interventions aimed at reducing fatigue and preserving manual dexterity among nurses. Practical strategies to achieve this could include the introduction of ergonomically designed workstations and tools that reduce physical strain, scheduled breaks to allow for recovery during shifts, and rotation of tasks to prevent repetitive strain on specific muscle groups. Additionally, incorporating hand exercises that focus on maintaining fine motor skills could be integrated into the daily routine of nurses, along with stress management programs to mitigate the psychological factors that contribute to physical fatigue. By implementing these strategies, healthcare facilities can help maintain the high levels of cognitive and physical performance required in emergency care settings, ultimately enhancing both patient care and nurse well-being.

## 6. Implications for Nursing Management

The findings of this study have significant implications for nursing management in emergency care settings, particularly in light of the declines in manual dexterity and the marked increase in perceived exertion following an 8-h work shift. Nursing managers need to critically evaluate work schedules and the allocation of duties to better support the physical and mental well-being of their staff. Optimizing shift patterns and incorporating sufficient rest periods could mitigate the decline in manual dexterity and increase in fatigue, enhancing overall nurse performance and patient care. Additionally, the study highlights the need for ergonomic improvements. Proactive steps should be taken to adjust the physical layout of work environments and the design of medical tools and devices. Ergonomically designed workspaces and tools can help reduce the physical strain on nurses, potentially preserving manual dexterity over longer periods and decreasing the risk of musculoskeletal injuries. Furthermore, regular training programs that focus on manual dexterity and strategies to manage physical exertion should be designed to be both practical and effective, particularly in the high-pressure environment of emergency nursing. These programs could include short, focused training sessions that can be integrated into the daily workflow, utilizing simulation-based exercises that replicate real-life emergency scenarios. Techniques for efficient task execution, such as using all surfaces of the hands and proper body mechanics, should be emphasized. Additionally, stress management techniques, such as breathing exercises and quick relaxation methods, could be incorporated into these programs to help nurses manage the physical and mental demands of their work. The use of ergonomic tools and equipment should also be part of the training, ensuring that nurses can effectively utilize these resources to reduce physical strain. Establishing comprehensive health and wellness programs that include physical fitness, stress management, and fatigue mitigation can further support nurses. Such programs would address not only the physical but also the psychological aspects of nursing work, helping to manage the increased perception of exertion and improve overall job satisfaction. Finally, nursing management should consider regular assessments of hand functionality and fatigue levels among nurses. These assessments can help identify when interventions are needed and track the effectiveness of implemented strategies over time. By addressing these areas, nursing management can significantly enhance the work environment and support systems for emergency service nurses, leading to improved health outcomes for both staff and patients.

## Figures and Tables

**Table 1:** Pre- and postshift hand function and perceived fatigue analyses.

	Preshift	Postshift	*t*-value	*p* value	Effect size (d)
MMDT (sec)	71.3 ± 2.1	74.9 ± 3.3	−6.297	<0.001	1.29 (large)
9-HPT (sec)	16.8 ± 1.4	17.6 ± 1.7	−8.920	<0.001	0.51 (small)
Handgrip strength (body mass kg·kg⁻^1^)	0.515 ± 0.023	0.512 ± 0.026	1.281	0.209	0.11 (trivial)
Pinch strength (body mass kg·kg⁻^1^)	0.102 ± 0.005	0.101 ± 0.006	1.187	0.244	0.12 (trivial)
Borg RPE	6.5 ± 0.6	12.1 ± 1.7	−19.927	<0.001	4.39 (nearly perfect)

*Note:* The values were presented as mean ± SD, and *p* < 0.05 was accepted as significant.

Abbreviations: 9-HPT, Nine-Hole Peg Test; Borg RPE, Borg Rating of Perceived Exertion Scale; MMDT, Minnesota Manual Dexterity Test.

**Table 2:** Preshift correlation matrix of hand function tests in emergency service nurses.

	9-HPT (sec)	Handgrip strength (body mass kg·kg⁻^1^)	Pinch strength (body mass kg·kg⁻^1^)
MMDT (sec)	0.744∗	0.154	0.141
9-HPT (sec)		0.156	0.263
Handgrip strength (body mass kg·kg⁻^1^)			0.794∗

Abbreviations: 9-HPT, Nine-Hole Peg Test; MMDT, Minnesota Manual Dexterity Test.

^∗^Correlation is significant at the 0.05 level (2-tailed).

**Table 3:** Correlations between percentage changes in hand function tests and perceived exertion in emergency service nurses.

	MMDT percentage change (5%)	9-HPT percentage change (4.5%)	Handgrip strength percentage change (0.53%)	Pinch strength percentage change (0.60%)
Borg RPE percentage change (86.5%)	0.545∗	0.562∗	0.413∗	0.267

Abbreviations: 9-HPT, Nine-Hole Peg Test; Borg RPE, Borg Rating of Perceived Exertion Scale; MMDT, Minnesota Manual Dexterity Test.

^∗^Correlation is significant at the 0.05 level (2-tailed).

## Data Availability

The data that support the findings of this study are available from the corresponding author upon reasonable request.
